# Brain volumetric reductions in patients with thrombotic thrombocytopenic purpura

**DOI:** 10.1371/journal.pone.0334903

**Published:** 2025-11-05

**Authors:** José Thiago de Souza de Castro, Simone Appenzeller, Letícia Rittner, Diedre Santos do Carmo, Fernanda A. Orsi, Fabiano Reis

**Affiliations:** 1 Department of Anesthesiology, Oncology and Radiology, Universidade Estadual de Campinas (UNICAMP), Campinas, São Paulo, Brazil; 2 Orthopedics and Rheumatology Unit of the Faculty of Medical Sciences, Universidade Estadual de Campinas (UNICAMP), Campinas, São Paulo, Brazil; 3 Medical Imaging Computing Lab (MICLab), Department of Computer Engineering and Automation, School of Electrical and Computer Engineering, Universidade Estadual de Campinas, Campinas, São Paulo, Brazil; 4 Department of Pathology, School of Medical Sciences, Universidade Estadual de Campinas (UNICAMP), Campinas, São Paulo, Brazil; Tekirdag Namik Kemal University: Tekirdag Namik Kemal Universitesi, TÜRKIYE

## Abstract

**Introduction:**

Thrombotic thrombocytopenic purpura (TTP) is a life-threatening disorder caused by a deficiency of a disintegrin and metalloproteinase with a thrombospondin type 1 motif, member 13 (ADAMTS13). The recognition of increased morbidity and mortality in patients after recovery suggests that TTP may become a chronic disease with possible multiple adverse outcomes throughout life. Neurological disorders may occur in the acute phase of TTP due to several pathophysiological mechanisms. These mechanisms include the formation of microvascular thrombosis in capillaries or small arteries of the central nervous system (CNS), leading to transient or permanent ischemic brain damage. However, the causes underlying chronic neurological involvement in TTP are not fully understood, particularly in long-term survivors. Therefore, the aim of this study is to evaluate brain volumetry in TTP patients in remission and to correlate it with the neurological involvement associated with the disease.

**Methodology:**

The study cohort includes 16 consecutive patients diagnosed with TTP between 1995 and 2016 at the Hospital de Clinicas, Universidade Estadual de Campinas (HC-UNICAMP), in Brazil. We performed MRI of the patients’ skull. Brain volume and parts of the central nervous system were analyzed and segmented using FreeSurfer.

**Results:**

The CNS volumetry of 16 TTP survivors was analyzed and compared with that of 20 age- and sex-matched controls. TTP survivors showed a reduction in intracranial volume (1,019 cubic liters (L^3^)) compared to the control group (1,280 L^3^), with a P value of 0.0003. The nucleus accumbens showed a significant reduction on the left side (469 mm^3^ in TTP survivors X 605 mm^3^ in controls, P = 0.02). The cerebellar cortex showed a reduction on the right side (44176.50 mm^3^ in TTP survivors X 47283.15 mm^3^ in control group, P = 0.05) and on the left side.

**Conclusion:**

Our study demonstrated a significant reduction in brain and nucleus accumbens volume in patients with TTP compared with a healthy control group. These findings suggest a possible lasting impact of TTP on the CNS and highlight the importance of continued monitoring and intervention to reduce neurological complications in patients after the acute phase of the disease. Future studies are needed to validate our results and elucidate the mechanisms underlying the brain changes observed in patients with TTP. Our findings provide valuable information for developing more effective treatment strategies and improving the quality of life of patients with TTP.

## Introduction

Thrombotic thrombocytopenic purpura (TTP) is a life-threatening disorder caused by a deficiency of a disintegrin and metalloproteinase with a thrombospondin type 1 motif, member 13 (ADAMTS13) [1,2]. The standard treatment for TTP is plasma exchange (PLEX) and corticosteroids, which have been shown to reduce mortality from 90 to 20% [3]. Despite treatment and clinical remission, a subset of TTP survivors may have late complications of the disease [4].

The recognition of increased morbidity and mortality in patients after recovery suggests that TTP may become a chronic disease with possible multiple adverse outcomes throughout life [5]. These outcomes include hypertension [6], obesity [5], cardiovascular disease, stroke [4,7,8], autoimmune disease [9], neurological disorders, such as cognitive dysfunction [10] and depression [6,11,12], reduced quality of life [13] and increased mortality [6].

Neurological disorders may occur in the acute phase of TTP [3,14,15] due to several pathophysiological mechanisms. These mechanisms include the formation of microvascular thrombosis in capillaries or small arteries of the central nervous system (CNS), leading to transient or permanent ischemic brain damage [4,7,16–21], hemorrhagic events, endothelial damage and posterior reversible encephalopathy syndrome (PRES) [22].

However, the causes underlying chronic neurological involvement in TTP are not fully understood [2,21–23]. The lack of understanding of the causes of long-term neurological disability may be due to the inability of routine CNS imaging to detect subtle brain damage.

Magnetic Resonance (MRI) volumetry is a technique that quantifies the volume of the CNS and has been used in other diseases [24] to demonstrate volumetric reduction of all or part of the CNS. Therefore, the aim of this study is to evaluate brain volumetry in TTP patients in remission and to correlate it with the neurological involvement associated with the disease. Therefore, this study aimed to assess brain volumetry in TTP patients during remission and explore its correlation with neurological involvement. While rituximab was not routinely used during part of the study period, it has since become a key element in the treatment of TTP—especially in refractory or relapsing cases—contributing to better long-term outcomes.

## Methodology

The study cohort includes 17 consecutive patients diagnosed with TTP between 1995 and 2016 at the Hospital de Clinicas, Universidade Estadual de Campinas (HC-UNICAMP), in Brazil. One participant underwent MRI, but due to artifacts the exam was discarded. Therefore, MRI data was obtained in 16 patients. The study was approved by the Institutional Ethics Committee of the School of Medical Sciences of the University of Campinas (CAAE 02465018.3.0000.5404). All participants received a consent form to participate in the study. The study obtained written informed consent from all participants prior to their inclusion. The consent form detailed the objectives, procedures, potential risks, and benefits of the study, ensuring that participants were fully informed before providing their agreement to participate. The study was designed and reported following key recommendations of the STROBE guidelines for observational studies.

Patients with a previous acute episode of TTP who were currently in clinical remission were included, these patients are referred to as TTP survivors. TTP was confirmed by a PLASMIC [19,25,26] score of 6 or 7 upon admission or ADAMTS13 activity below 10%. Before 2010, ADAMTS13 was not available in the Hospital, therefore patients were diagnosed and treated for TTP if they presented on admission with platelet count < 150 x 10^9^/L, hemolytic microangiopathy (hemoglobin level <10 g/dL long with schistocytes on peripheral blood smear), clinical course consistent with TTP (response to plasma exchange therapy), and the absence of alternative thrombotic microangiopathies such as transplant associated microangiopathy or atypical hemolytic uremic syndrome were excluded from the study. To confirm the diagnosis of those treated before 2010 (n = 5 patients), we calculated their PLASMIC score using the data from the admission.

We excluded patients with pre-TTP neurological disease, and contraindications to MRI (claustrophobia, pacemaker, aneurysm clip, and other conditions that pose a risk to patient safety). Clinical and laboratory data of the acute TTP episode were retrospectively retrieved from the patients’ medical records, based on information written by the medical team and test results.

All patients included in the study were in clinical remission of TTP and were followed in the outpatient clinic. They were recruited during the outpatient visit. Brain volumetric analysis was performed using MRI volumetry and automated segmentation software (FreeSurfer). We also obtained brain MRI volumetry from a healthy control group of similar age and sex, with the aim of comparing the cerebral involvement of TTP patients with controls. The controls consist of selected T1-weighted MRI scans of the head of healthy subjects, previously collected in the same MRI scanner at HC-UNICAMP, for control purposes, with Ethics Review Board approval.

The recruitment of participants took place between 01/02/2019 (DD/MM/YYYY), and 28/02/2019. Imaging assessments were conducted between 01/03/2019, and 30/06/2019, while medical records were retrospectively accessed from 01/02/2019, to 30/06/2019, for clinical data analysis.

Although not all items from the STROBE checklist are explicitly detailed, the manuscript includes key elements such as participant selection, data sources, outcome measures, and statistical methods, ensuring greater transparency and reproducibility.

### Image acquisition

The MRI was carried out using Philips® Achieva 3T equipment, at HC-UNICAMP, without contrast. The protocol used included T1-weighted spin echo, T2-weighted turbo spin echo, T2-weighted ﬂuid attenuated inversion recovery (T2-FLAIR), diffusion-weighted imaging (DWI), Susceptibility weighted imaging (SWI) and Voxel-Based Morphometry (VBM). The MR images (T1, T2, T2-FLAIR, SWI, and DWI) were analyzed by an experienced neuroradiologist.

### Segmentation, image analysis and control group

The segmentation of brain structures visible in neuroimaging, such as T1-weighted MRI, reveals their morphological properties and volumes. These can be associated with diseases and aid in diagnosis and patient follow-up [27]. However, manual delineation of brain structures, especially in large neuroimaging datasets, is a time-consuming and expensive task, which has motivated the development of many automated segmentation methods. In this study, we used FreeSurfer [28], an established automated brain structure segmentation pipeline. FreeSurfer is a collection of methods developed over the last decades to provide robust morphometric analysis of the brain in T1-weighted images. Its pipeline consists of several steps: preprocessing including motion correction, intensity normalization, and skull stripping; followed by registration to a standardized atlas; gray matter, white matter, and cerebrospinal fluid; cortical surface reconstruction; topology correction; and finally, subcortical and white matter segmentation. We used version 7.4.1, released in June 2023.

The brain regions analyzed in this study included subcortical and infratentorial areas related to motor, emotional, cognitive, and visual functions. We chose these regions based on prior evidence linking neuropsychiatric symptoms to vascular and autoimmune diseases, and considering their clinical relevance to symptoms frequently reported by TTP survivors. While significant differences emerged in some regions—such as the nucleus accumbens, putamen, and optic chiasm—others, like the hippocampus and amygdala, showed no notable differences between groups.

Our control group consisted of healthy individuals recruited from the same hospital and during the same timeframe as the TTP patients. These volunteers had no history of neurological or psychiatric disorders, and were closely matched to the TTP group in terms of age and sex. Each control participant underwent the same MRI protocol and was screened carefully to rule out medical conditions known to influence brain volumes, such as diabetes, hypertension, stroke, or autoimmune disorders.

All MRI examinations were conducted in 2019 using the same 3T Philips Achieva scanner (Philips Medical Systems, Best, Netherlands) located at the Hospital de Clínicas, University of Campinas (HC-UNICAMP). Identical scanning parameters were consistently applied to ensure uniformity across all participants.

### Statistical analysis

Descriptive measures (mean, standard deviation, minimum, median, and maximum) of numerical variables were presented to characterize the sample. The Mann-Whitney test was used to compare MRI measurements between groups. The significance level adopted for the study was 5%. We used the SAS System for Windows (Statistical Analysis System), version 9.4. SAS Institute Inc, 2002–2012, Cary, NC, USA.

## Results

The mean age of TTP survivors at the time of examination was 40 years (Table 1). Of the 16 survivors, 15 were women. The median time between the first episode of TTP and MRI was 83 months, ranging from 3 to 177 months. All survivors had only 1 episode of TTP, except for one patient who had 4 episodes. Most survivors were presented with neurological symptoms in the acute phase of TTP (93.8%), as detailed in Table 2. The median ADAMTS13 activity upon diagnosis was 4.3% and the PLASMIC score was 6.3. All survivors were treated with plasma exchange and corticosteroids in the acute phase. Vincristine was used in 56.3% and rituximab in 12.5% of TTP patients.

**Table 1:** Baseline characteristics of the 16 patients.

Feature	Result
Age on the date of MRI (years), average (range)	40,75 (18-62)
Woman, n (%)	15 (93,8)
Ethnic group:	
White, n (%)	10 (62,5)
Black, n (%)	5 (31,3)
Indigenous, n (%)	1 (6,3)
Period between first episode of TTP and MRI (months)	83 (3-177)
Abnormal results on MRI	6 Y, 9 N
Number of TTP episodes on the date of MRI, average (range)	1,2 (1-4)
Neurological manifestation during the hospitalization, n (%)	15 (93,8)
Pregnancy or puerperium at TTP diagnosis, n (%)	5 (33,3)
ADAMTS13 at diagnosis, average (range)	4,3% (0,00% − 9,07%)
PLASMIC score at diagnosis, average (range)	6,25 (6-7)
Plasma exchange or corticosteroid during hospitalization, n (%)	16 (100)
Vincristine during hospitalization, n (%)	9 (56,3)
Rituximab during hospitalization, n (%)	2 (12,5)
Splenectomy during hospitalization, n (%)	3 (18,7)

**Abbreviations:** MRI: Magnetic Resonance Image; N: number; F: female; M: male; TTP: thrombotic thrombocytopenic purpura; NA: not applicable. ADAMTS13: Disintegrant And Metalloprotease with eight Thrombo Spondin-1-like. BMI: body mass index.

**Table 2:** Neurological manifestations in the 16 patients at hospitalization.

Feature	Result
Headache, n (%)	12 (75)
Confusion, n (%)	10 (62,5)
Stupor or Comatose, n (%)	9 (56,3)
Sensorimotor loss, n (%)	7 (43,75)
Seizures, n (%)	5 (33,3)
Personality Changes, n (%)	4 (25)
No neurological symptoms, n (%)	1 (6,25)

**Abbreviations:** N: number.

**Fig 1:**
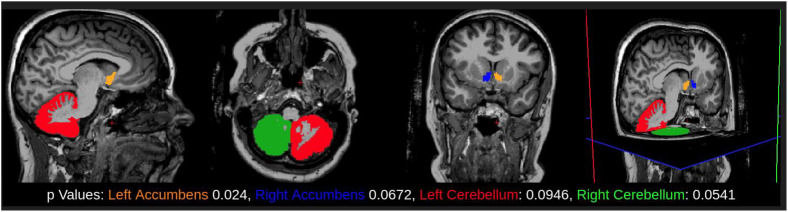
Highlights the anatomical sites with significant volume reduction or a tendency towards volume reduction, found in this study.

The CNS volumetry of 16 TTP survivors was analyzed and compared with that of 20 age- and sex-matched controls. The volumetry results are presented in Table 3. TTP survivors showed a reduction in intracranial volume (1,019 cubic liters (L^3^) compared to the control group (1,280 L^3^), with a P value of 0.0003. The nucleus accumbens showed a significant reduction on the left side (469 mm3 in TTP survivors X 605 mm^3^ in controls, P = 0.02) and a non-significant trend toward volume reduction on the right side (499 mm^3^ in TTP survivors X 608 mm^3^ in controls, P = 0.06) – Fig 1. The cerebellar cortex showed a reduction on the right side (44176.50 mm^3^ in TTP survivors X 47283.15 mm^3^ in control group, P = 0.05) and on the left side.

**Table 3:** Volumetry of the central nervous system and cerebral structures, using FreeSurfer.

Region	Controls (N = 20)	TTP Survivors (N = 16)	Total (N = 36)	P-Value	Difference (95% CI)
Estimated Total Intra Cranial Vol (Mean ± SD (L^3^))	1.267 ± 1.731	1.038 ± 1.407	1.165 ± 1.952	0.0003	
Estimated Total Intra Cranial Vol (Median (min-max)) L^3^	1.280 (0.835-1.5478)	1.019 (0.850-1.403)	1.156 (0.835-1.548)	0.0003	
Optic Chiasm (Mean ± SD (N)) mm^3^	138.03 ± 46.00	109.83 ± 47.36	125.50 ± 48.08	0.03	–34.3 (–63.7 to –5.0)
Optic Chiasm (Median (min-max)) mm^3^	143.65 (21.60-204.60)	107.35 (33.10-214.80)	134.70 (21.60-214.80)	0.03	
Left Accumbens area (Mean ± SD) mm^3^	599.02 ± 113.97	501.67 ± 130.34	555.75 ± 129.38	0.02	–97.4 (–180.1 to –14.6)
Left Accumbens area (Median (min-max)) mm^3^	605.40 (375.10-836.00)	469.15 (261.20-806.90)	567.75 (261.20-836.00)	0.02	
Right Accumbens area (Mean ± SD) mm^3^	609.79 ± 117.74	539.53 ± 135.58	578.56 ± 129.06	0.06	
Right Accumbens area (Median (min-max)) mm^3^	608.70 (397.60-817.40)	499.85 (355.50-811.80)	549.05 (355.50-817.40)	0.06	
Right Cerebellum Cortex (Mean ± SD) mm^3^	47680.43 ± 4440.02	45607.72 ± 5403.77	46759.23 ± 4930.26	0.05	
Right Cerebellum Cortex (Median (min-max)) mm^3^	47283.15 (37738.40-54124.90)	44176.50 (39219.00-61802.00)	45292.30 (37738.40-61802.00)	0.05	
Left Cerebellum Cortex (Mean ± SD) mm^3^	47213.41 ± 4325.30	45080.51 ± 4498.24	46265.46 ± 4470.24	0.09	
Left Cerebellum Cortex (Median (min-max)) mm^3^	46377.40 (37609.90-54547.10)	44287.30 (38848.20-56101.30)	45440.75 (37609.90-56101.30)	0.09	

**Abbreviations:** TTP: thrombotic thrombocytopenic purpura; N: number; SD: standard deviation.

Note: Differences and 95% confidence intervals were calculated for the comparisons between TTP survivors and controls using independent t-tests with unequal variances.

Along with reporting p-values, we also calculated 95% confidence intervals (CI) for key group comparisons. We found statistically significant mean reductions of –97 mm^3^ in the left nucleus accumbens (95% CI: –180 to –15 mm^3^; p = 0.025) and –34 mm^3^ in the optic chiasm (95% CI: –64 to –5 mm^3^; p = 0.027). Additionally, the left putamen showed an increase of +1017 mm^3^, but this difference was not statistically significant (95% CI: –750 to +2784 mm^3^; p = 0.19).

To better control for variability in overall brain size, ANCOVA analyses were conducted using total intracranial volume (TIV) as a covariate. Notably, the mid-anterior corpus callosum remained significantly smaller even after this adjustment (p = 0.0188), suggesting a specific regional change rather than general brain atrophy.

## Discussion

In this study, we performed MRI with an advanced technique, volumetry, with the aid of software, to analyze the CNS in a chronic phase of the disease. Despite a small group of patients, we observed a statistically significant reduction in brain volume, and nucleus accumbens. The reduction in total intracranial volume in TTP patients was 20,34% on average, and the nucleus accumbens bilaterally was 13,86% smaller.

The pathophysiology of TTP is directly linked to a deficiency or dysfunction of ADAMTS13. This enzyme plays an important role in blood coagulation, acting in the cleavage of von Willebrand multimer (vWF). In normal situations, vWF facilitates the adherence of platelets to sites of vascular injury, thus helping to form a clot to stop bleeding. However, in TTP, due to deficiency or the presence of antibodies that inhibit ADAMTS13 results in the persistence of large vWF multimers. The presence of these multimers promotes platelet aggregation and the widespread formation of vascular microthrombi, particularly affecting the brain. The accumulation of microthrombi in cerebral vessels can cause structural and functional lesions, which is one of the possible explanations for the reduction in brain volume observed in patients with TTP [2,29,30].

Besides, approximately a quarter of patients with TTP have changes consistent with posterior reversible encephalopathy syndrome (PRES) being present in half of these cases [16]. Although most cases are completely reversible, some patients with PRES may have residual sequelae and significant residual lesions in MRI follow up [31]. In some cases, particularly during the acute phase of TTP, severe thrombocytopenia may lead to brain hemorrhages, which in turn may result in chronic brain damage [32].

The cerebellum plays a significant role in cognitive and affective processing [33–35]. It has been proposed that the cerebellum contributes to cognition and motor functioning through the formation of internal models that support the coordination of behavior and skill learning. As a new model is formed, it can shape cortical representations so that once the internal model of behavior is acquired, it can be stored in the cortex and accessed flexibly [36].

We identified atrophy of the nucleus accumbens bilaterally, something already identified and related to melancholic depression [37–39] and in Parkinson’s disease [38].

The reduction in nucleus accumbens volume observed among TTP survivors may indicate disruption of reward and motivational pathways closely associated with emotional regulation. Given that this subcortical region is central to emotional and motivational processing, these structural alterations might help explain the frequent complaints of depressive symptoms and decreased motivation in these patients.

Of note, after adjusting for total intracranial volume, the mid-anterior corpus callosum emerged as the sole region with a statistically significant reduction in volume (p = 0.0188). The corpus callosum is crucial for efficient interhemispheric communication and is commonly affected in diseases involving chronic microvascular injury or demyelinating processes. As far as we know, this study is the first to report structural changes in the corpus callosum specifically in TTP survivors, possibly highlighting subtle, persistent damage in these individuals.

Previous studies have shown that TTP survivors have more neurological symptoms, strokes, and cognitive deficits than the general population at similar ages [4]. The underlying mechanisms are not yet understood. Here we have demonstrated that TTP survivors have a reduction in CNS volume, which may be the cause of chronic neurological involvement. Preventing brain volume loss could potentially reduce complications associated with TTP and enhance survivors’ long-term quality of life. While the exact mechanisms behind this volumetric reduction are still unclear, persistent endothelial dysfunction or ongoing subclinical neuroinflammation might be contributing factors. Interventions such as prolonged immunomodulatory treatments, neuroprotective approaches, and early detection of cognitive changes through imaging and neuropsychological evaluations may help mitigate these outcomes.

The brain regions analyzed in this study were chosen based on their functional importance and prior evidence from disorders involving cerebrovascular compromise. For example, the nucleus accumbens plays a critical role in emotional regulation and motivation, while the putamen and optic chiasm are key structures involved in motor control and visual pathways, respectively. Although we also assessed regions like the hippocampus and amygdala—central to memory and emotion processing—we found no significant volume differences between TTP survivors and the control group in these areas. Larger studies in the future might help clarify whether subtle changes occur in these structures.

Although our primary focus was on subcortical and infratentorial areas associated with motor, visual, and emotional functions, we also included cortical regions commonly linked to cognitive impairment, such as the hippocampus, amygdala, and temporal lobes. In this particular group of TTP survivors, however, no significant differences emerged in these areas compared to controls. Given the established relationship between cognitive dysfunction and thrombotic microangiopathies, these regions certainly deserve attention in future investigations with larger sample sizes. Studies like the ARIC neurocognitive cohort have identified substantial volumetric differences in individuals with mild cognitive impairment (MCI), especially in temporal regions [38], suggesting possible overlaps or distinctions relevant to chronic TTP.

The relationship between cognitive impairment and volumetric brain changes has been well documented. For instance, findings from the ARIC study highlight significant differences in hippocampal and thalamic volumes among people with MCI [38]. While our analysis included these regions, we did not observe statistically significant changes, possibly indicating that the neuroanatomical pattern of volumetric reduction in TTP survivors differs from classic MCI. This discrepancy might reflect unique microvascular mechanisms characteristic of TTP.

Our study has some limitations that should be discussed. This is a single center study and the same size is small, which is justified by the rarity of the disease. Due to the small sample size, it was not possible to establish a relationship between the volumetric reduction of the CNS and other important factors, such as ADAMTS13 deficiency, comorbidities, presence or absence of anti-ADAMTS13 antibodies, cognitive impairment, depression or other neurological involvement. In addition, as a retrospective study, the timing of volume reduction after acute TTP could not be demonstrated. Finally, although the control group was matched for age and sex to the TTP survivors, there may be other confounding variables that could influence the results, such as pre-existing comorbidities or medication use. We recognize that factors such as pre-existing medical conditions (e.g., hypertension), long-term immunosuppressive medication use, and specific aspects of TTP itself—like ADAMTS13 activity and antibody status—could have impacted brain volume measurements. Due to the retrospective nature and limited sample size of our study, we were unable to statistically control for these potential confounders. Future prospective research with larger patient groups is needed to better understand and clarify these relationships.

## Conclusions

In conclusion, our study demonstrated a significant reduction in brain and nucleus accumbens volume in patients with TTP compared with a healthy control group. These findings suggest a possible lasting impact of TTP on the CNS and highlight the importance of continued monitoring and intervention to reduce neurological complications in patients after the acute phase of the disease. Once we adjusted for total intracranial volume, the mid-anterior corpus callosum was the only region that still showed a significant volume reduction. This structure plays a crucial role in connecting the brain hemispheres, and its selective atrophy observed here may reflect subtle, ongoing microvascular damage in TTP survivors. As far as we are aware, this finding has not been previously reported, and thus it represents a promising area for future studies, potentially serving as a biomarker for chronic microvascular injury in these patients. Future studies are needed to validate our results and elucidate the mechanisms underlying the brain changes observed in patients with TTP. Our findings provide valuable information for developing more effective treatment strategies and improving the quality of life of patients with TTP.

## Supporting information

S1 File Control group. (XLSX)

S2 File TTP group. (XLSX)
